# Inhibitory Effects of a Reengineered Anthrax Toxin on Canine Oral Mucosal Melanomas

**DOI:** 10.3390/toxins12030157

**Published:** 2020-03-02

**Authors:** Adriana Tomoko Nishiya, Marcia Kazumi Nagamine, Ivone Izabel Mackowiak da Fonseca, Andrea Caringi Miraldo, Nayra Villar Scattone, José Luiz Guerra, José Guilherme Xavier, Mário Santos, Cristina Oliveira Massoco de Salles Gomes, Jerrold Michael Ward, Shihui Liu, Stephen Howard Leppla, Thomas Henrik Bugge, Maria Lucia Zaidan Dagli

**Affiliations:** 1 Department of Pathology, School of Veterinary Medicine and Animal Science, University of Sao Paulo, Sao Paulo 05508-270, SP, Brazil; adriananishiya@usp.br (A.T.N.); mknagamine@gmail.com (M.K.N.); ivonemackowiak@gmail.com (I.I.M.d.F.); andreia.caringi@gmail.com (A.C.M.); nayra.villar@gmail.com (N.V.S.); guerra@usp.br (J.L.G.); cmassoco@usp.br (C.O.M.d.S.G.); 2 Rous Animal Pathology, Private Veterinary Pathology Services. Av. Lacerda Franco 127, Sao Paulo 01536-000, SP, Brazil; xavier2126@gmail.com (J.G.X.); msantos@gmail.com (M.S.); 3 Global Vet Pathology, Montgomery Village, MD 20886, USA; veterinarypathology@gmail.com; 4 Aging Institute and Division of Infectious Diseases, Department of Medicine, University of Pittsburg, Pittsburgh, PA 15261, USA; SHL176@pitt.edu; 5 Microbial Pathogenesis Section, Laboratory of Parasitic Diseases, National Institute of Allergy and Infectious Diseases, National Institutes of Health, Bethesda, MD 20892, USA; sleppla@niaid.nih.gov; 6 Proteases & Tissue Remodeling Section, National Institute of Dental and Craniofacial Research, NIH, Bethesda, MD 20892, USA; thomas.bugge@gmail.com

**Keywords:** toxin, oral melanoma, dog, *Bacillus anthracis*, anthrax

## Abstract

Canine oral mucosal melanomas (OMM) are the most common oral malignancy in dogs and few treatments are available. Thus, new treatment modalities are needed for this disease. *Bacillus anthracis* (anthrax) toxin has been reengineered to target tumor cells that express urokinase plasminogen activator (uPA) and metalloproteinases (MMP-2), and has shown antineoplastic effects both, in vitro and in vivo. This study aimed to evaluate the effects of a reengineered anthrax toxin on canine OMM. Five dogs bearing OMM without lung metastasis were included in the clinical study. Tumor tissue was analyzed by immunohistochemistry for expression of uPA, uPA receptor, MMP-2, MT1-MMP and TIMP-2. Animals received either three or six intratumoral injections of the reengineered anthrax toxin prior to surgical tumor excision. OMM samples from the five dogs were positive for all antibodies. After intratumoral treatment, all dogs showed stable disease according to the canine Response Evaluation Criteria in Solid Tumors (cRECIST), and tumors had decreased bleeding. Histopathology has shown necrosis of tumor cells and blood vessel walls after treatment. No significant systemic side effects were noted. In conclusion, the reengineered anthrax toxin exerted inhibitory effects when administered intratumorally, and systemic administration of this toxin is a promising therapy for canine OMM.

## 1. Introduction

Oral mucosal melanomas (OMM) are the most common oral malignancy in dogs [1,2]. OMM are characterized by local infiltration and metastasis to regional lymph nodes (11.4–53% of cases) [3,4] and lungs (23–27%). Recurrence rates are between 3.2 and 10% and the median survival after diagnosis ranges from 65 to 1020 days [4,5,6,7]. The common treatment modalities for OMM include surgery, radiation therapy, chemotherapy and immunotherapy [2]. Novel strategies, like vaccines, are commercially available and have some inhibitory effects [8,9]. Some new therapies, such as nanotechnology-based immunotherapy [10], electro-chemotherapy [11], and toceranib phosphate (Palladia^®^), alone or in combination [12], have also been reported, but only partial responses were observed. Thus, new treatments for OMM are needed.

Here we report the results of a clinical study on the effects of a reengineered anthrax toxin on canine OMM. The anthrax (*Bacillus anthracis*) toxin is composed of three individual proteins — lethal factor (LF), edema factor (EF), and protective antigen (PA). None of the three subunits displays any biological effects in animals when administered alone, but PA combined with EF or LF cause skin edema and death, respectively, in animals [13,14].

Since 1999, the group of Liu, Leppla and Bugge from the National Institutes of Health (NIH) has been working on the potential of the anthrax toxin to treat cancer [13,15]. Their goal was to modify the anthrax toxin components so that it could use resources from the tumor cells to selectively kill them.

To kill host cells, the PA native anthrax toxin subunit binds to endothelial cell surface receptors called Tumor Endothelial Marker 8 (TEM8 or ANTXR1) or Capillary Morphogenesis Gene 2 (CMG2 or ANTXR2) [16], and is subsequently cleaved by a furin protease on the cell membrane surface [17]. This cleavage generates a 63-kDa C-terminal fragment that subsequently forms a PA heptamer that binds and translocates up to three molecules of LF or EF into the cytosol. EF is a potent adenylate cyclase protein that kills cells by raising cyclic adenosine monophosphate (cAMP) levels, whereas LF is a metalloproteinase that cleaves and inactivates mitogen-activated protein kinase kinases (MEKs), thereby blocking the extracellular signal-regulated kinase (ERK)/mitogen-activated protein kinase (MAPK) pathway [18].

To selectively kill tumor cells, the reengineered anthrax toxin targets three over-expressed proteins: The urokinase-type plasminogen activator (uPA) and its receptor (uPAR), and the metalloproteinases (MMPs). Thus, mutated anthrax toxin-protective antigen (PA) proteins, in which the furin cleavage site is replaced by sequences cleaved specifically by uPA, and modified PA proteins, in which the furin protease cleavage site is replaced by sequences selectively cleaved by MMPs, were developed. The high cytotoxicity of anthrax toxin and the overexpression of uPA/uPAR and MMP in various tumor types favored the construction of mutated versions of PA [18]. To further enhance tumor specificity, inter-complementing *Bacillus anthracis* toxin was engineered to be dependent on the activation of uPAs and MMP. The inter-complementing toxin consists of PA variants PA-U2-R200A and PA-L1-I210A, which cause cell death by disruption of the MAPK signaling pathway when associated with LF. In vivo, this association showed the best therapeutic index for xenografted human melanomas and carcinomas [19,20]. Nevertheless, the efficacy of the engineered anthrax toxin has never been tested on canine tumors.

Proteinases urokinase (uPA) and metalloproteinases (MMPs) are overexpressed in a variety of tumor cells and are rarely present in physiologically normal cells [21]. Canine melanocytic tumors showed high MMP-2 activity [22] and melanoma cell lines express MMP-9 [23]. To date, uPA and uPAR have not been studied in canine OMM, but expression occurs throughout the canine genitourinary tract [24] and in canine mammary tumors [25]. Dogs are considered good models for human cancers, and here, we test the effects of a re-engineered anthrax toxin on canine OMM.

## 2. Results

### 2.1. Clinical and Histological Characteristics

Five dogs (numbered 1 to 5) with spontaneous OMM were included in the study and the dogs’ and OMM characteristics can be seen in Table 1. Four animals were male and age and weight ranged from 11 to 16 years and 5, to 33,3 Kg, respectively. OMM staging ranged from I/IV (dog 5) to III/IV (dogs 1–4) and tumors were mostly located in the maxilla (3/5), followed by mandible (1/5), and hard palate (1/5). Animals 1–4 presented lymph node metastasis. Initial tumor volume ranged from 228 to 18602 mm³ before treatment.

### 2.2. Evaluation of the Clinical Response of Canine OMM to the Reengineered Anthrax Toxin

The five dogs were treated with intratumoral doses of the reengineered anthrax toxin and LF for assessing the efficacy of in vivo treatment. Dogs 1 and 2 received 6 injections of the toxin in 14 days of treatment whereas dogs 3, 4, and 5 received 3 inoculations of the toxin every other day, within 7 days. Variation in treatments between dogs was based on clinical findings in the first dogs injected. Dogs 1 and 2 were treated with 6 intratumoral applications of the anthrax toxin (during 14 days), while dogs 3, 4 and 5 were treated with only 2 doses, during 7 days. Dogs 1 and 2 were treated first, and at the 9th day after treatment an increase in tumor volumes was observed, probably due to the production of antibodies against the toxin, as mentioned by Liu et al., 2016 [27]. This effect could impair the response to treatment, causing the increase in volume and discomfort to the animal. Therefore, dogs 3, 4, and 5 received only 3 intratumoral injections of the toxin.

**Figure 1:**
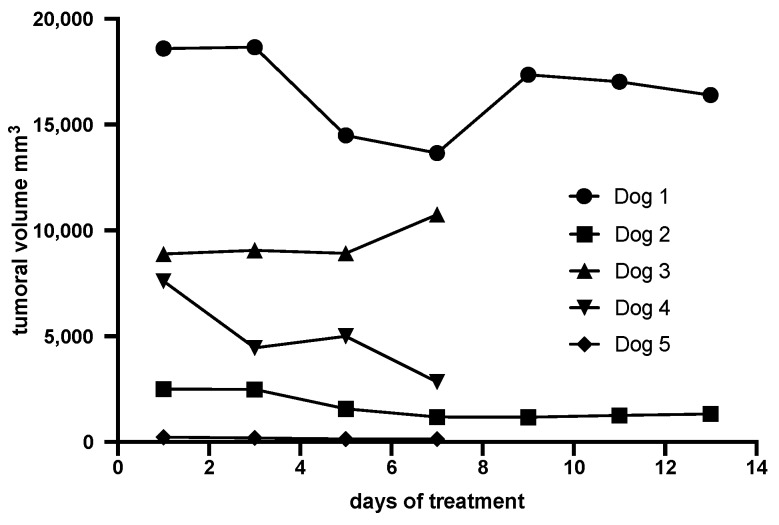
Tumoral volume of canine OMM in 5 dogs after intratumoral treatment with the reengineered anthrax toxin.

The clinical response to the reengineered toxin treatment was evaluated during the first seven or 14 days before surgery. There was no disease progression; 4 dogs showed tumor reduction varying from 12 to 63% (Table 2, Figure 1). One dog (dog 3) showed 20% increase in the tumor, due to local edema.

Clinical examination revealed enlarged sentinel lymph nodes after the first injection of the toxin and decreased tumor bleeding after 3 or 6 injections in all dogs. The toxin was generally well tolerated; local facial edema and ulceration of oral mucosa were observed only in dog 2. The treatment with the reengineered anthrax toxin caused no adverse effects like weight loss or significant changes in blood parameters, including packet cell volume (Ht), total leukocytes (Leuko), serum alanine aminotransferase (ALT), alkaline phosphatase (ALP), urea (Urea), creatinine (Creat), and platelets (Plat) (Table 3). No significant systemic side effects were noted in any animals; dogs 2 and 5 were still alive 532, and 288 days, respectively, after the intratumoral toxin treatment followed by surgery.

### 2.3. Histopathology, Immunostaining, and Cell Proliferation of Canine OMM Before and After Treatment with the Reengineered Anthrax Toxin

Histopathological diagnosis of OMM are presented in Table 1 and Table 4. Dog 1 had an amelanotic melanoma, while dogs 2, 3, 4, and 5 had melanotic OMM.

**Figure 2:**
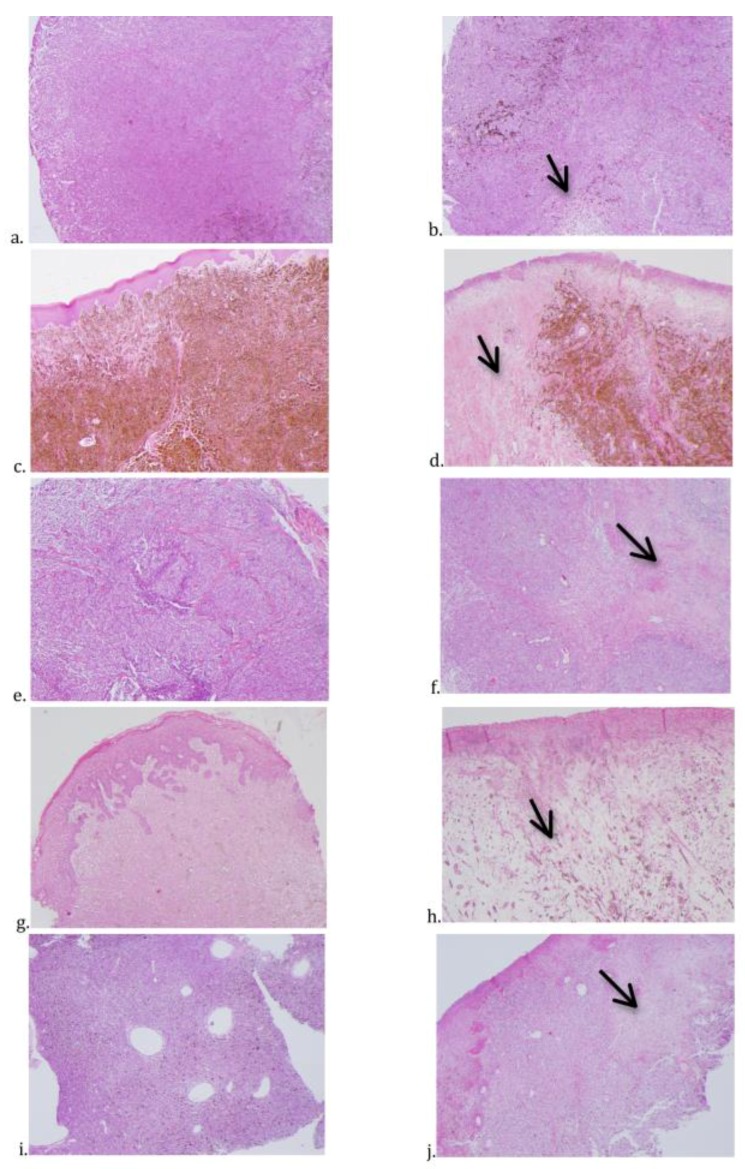
Photomicrographs of canine OMM biopsy samples from dogs 1 (**a,b**), 2 (**c,d**), 3 (**e,f**), 4 (**g,h**) and 5 (**i,j**), obtained before (**a,c,e,g,i**) and after (**b,d,f,h,j**) the intratumoral treatment with the reengineered anthrax toxin. In samples obtained after the treatment, it is possible to see necrotic areas (arrows), as well as edema and hemorrhage. All photomicrographs were obtained in a stereo microscope (Nikon^®^), with a magnification of 5×. (H&E, Scale bar = 0.5 mm.).

**Figure 3:**
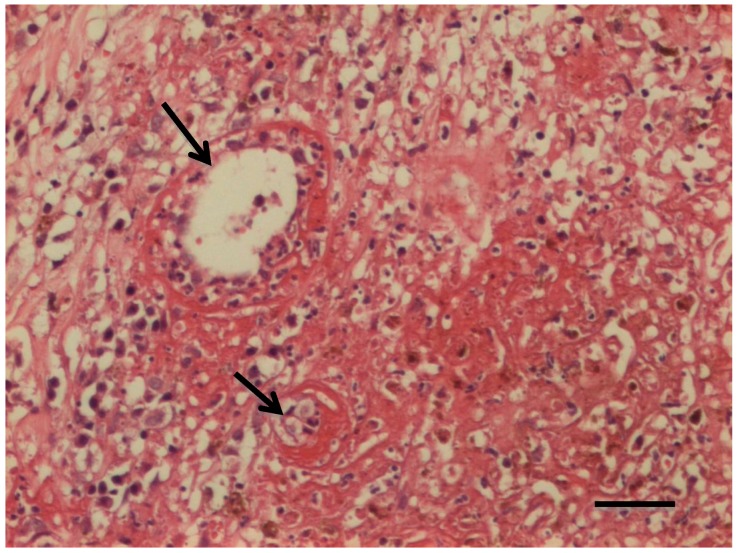
Detail of histopathological sample of OMM from dog 4, showing necrosis of the blood vessel walls (arrows). (H&E, Scale bar = 100 μm.).

The histological changes observed after treatment with the reengineered anthrax toxin were mainly inflammation infiltrating the tumor masses, with predominance of lymphocytes in melanomas of dogs 1 to 4 and of neutrophils in dog 5. Areas of necrosis, hemorrhage, and edema were identified in histological sections from dogs 1 to 4 (Table 5, Figure 2). Only dog 5 had no lymph node metastasis. Histopathology of tumors from animals 3, 4, and 5 showed necrosis in blood vessel walls after intratumoral treatment with the reengineered anthrax toxin (Figure 3).

Cell proliferation of the toxin treated tumors was determined by immunostaining with the cell proliferation marker Ki-67. Three animals (dogs 2, 3, and 4), had a decreased Ki-67 index after toxin treatment (Table 5).

Immunostaining for uPA, uPAR, MMP-2, MT1-MMP, and TIMP-2 was positive for most dogs, except MT1-MMP for dog 1 (Table 6).

## 3. Discussion

The treatment of both human and animal cancers still remains one of the greatest challenges to science. Among domestic animals, and similarly to humans, dogs are living longer and are the most affected by several types of neoplasia.

Murine cancer models have been useful for analyzing the biology of pathways involved in cancer initiation, promotion, and progression. However, they often lack features that define cancer in humans, including long periods of latency, genomic instability, tumor cell heterogeneity, and its surrounding microenvironment. More importantly, the complex biology of cancer recurrence and the development of metastases are not adequately reproduced in the conventional models of laboratory animals used in the development of antineoplastic drugs. Due to these shortcomings, the development and approval of new cancer drugs has been a lengthy and expensive process, and therefore, additional models that better represent human disease are needed.

In this scenario, veterinary oncology has recently gained significant prominence in the scientific community, particularly canine comparative oncology. Tumors of dogs and humans have several similarities, including histological appearance, genetic alterations, molecular targets, biological behavior, and responses to conventional therapies [30]. In fact, several research groups around the world have turned their attention to spontaneous cancers of dogs as models of human neoplasms, since the dog genome published in 2005 [31] showed many similarities between the two species.

Several types of neoplasms, including, among others, melanomas, share indisputable similarities with the corresponding ones in humans. [32,33]

Canine OMM is an aggressive disease, and this study aimed to evaluate a new possible targeted therapy for these tumors. Evidence for the antineoplastic action of the re-engineered anthrax toxin in experimental studies has prompted us to begin testing on canine OMM.

*Bacillus anthracis* toxin has been studied since 1955 on the evidence of high lethality in mice and guinea pigs. The toxin consists of three subunits: A protective antigen (PA), an edema factor (EF) and lethal factor (LF). PA is cleaved by furin proteases on the cell membrane forming an active heptamer (PA63) bound to the *Bacillus anthracis* toxin receptor (TEM8 or CMG2). This association forms a channel through which EF and LF can translocate into the cytosol, causing cell death [13,17,27,34,35,36] by increasing levels of intracellular cAMP, or blocking proteins of the MAPK signaling pathway, respectively [5,37,38].

Evidence of the presence of metalloproteinases and urokinases mainly in neoplastic cells [19,39,40] has opened the possibility of reengineering *B. anthracis* toxin (mutated PA) to be activated by uPA and MMP proteases. In the early 2000s, Liu and colleagues synthesized PA variants PA-L1-I210A and PA-U2-R200A, modifying the site of furin action to be cleaved specifically by MMP, and uPA, respectively, on the cell membrane [14,18,27,41]. This modified *Bacillus anthracis* toxin was selective to cells that express both proteases in their cell membrane and had diminished off-target cytotoxicity when compared to native *Bacillus anthracis* toxin [18] Since then, cytotoxicity tests and xenotransplantation studies, using mouse models, have been performed on human cell lines of head and neck carcinomas, melanomas and lung, uterine, and intestinal carcinomas [18,39,40,42], as well as in murine tumors, such as fibrosarcomas, Lewis carcinomas, and melanomas [19,36].

In this study, animals were selected according to pre-defined criteria, and biopsies of their oral lesions were taken to confirm the diagnosis of melanoma. The dogs received intratumoral injections and tumor progression was assessed for 14 days, followed by surgical resection of the lesions for ethical reasons.

We decided to first inject the re-engineered anthrax toxin intratumorally to assess the clinical response of dogs, before systemically administering the toxin. In an experimental study, Peters and colleagues [43] found 32–87% tumor reduction in B16 melanomas transplanted into BL6 mice after treatment with an engineered anthrax toxin, but complete tumor remission was not achieved. Based on the cRECIST v 1.0 [29] all five dogs in our study showed stable disease when tumor diameters were considered. Four dogs had 12 to 63% reduction in OMM volume. According to literature data, OMM, in general, have very poor prognosis, with the possibility to metastasize to the lungs and lymph nodes. The overall median survival time is less than 36 months, depending on the size, histopathology of the tumor, and the stage of the disease [2]. Therefore, it is difficult to predict what would happen with the present OMM cases without treatment.

This study did not aim to evaluate the effect of the treatment on the survival. However, it has been possible to detect that, even with a short period of intratumoral treatment with the anthrax toxin, some clinical benefits were observed. The diseases became stable according to cRECIST and there was decreased bleeding.

Systemic parenteral administration of *Bacillus anthracis* toxin has been used in several mouse studies and provides adequate diffusion of toxin throughout the tumor tissue, mainly reaching CMG2 receptors in endothelial cells of the capillaries, venules, and arterioles [36]. Necrosis of blood-vessel endothelial cells was also observed in our canine melanoma samples after intratumoral toxin injection. However, intratumoral administration provides irregular distribution by passive or facilitated diffusion of proteins and the toxin may not reach tumor endothelial cells, which is the main site of action of anthrax toxin. Inhibition of the MAPK pathway by LF in endothelial cells does not promote apoptosis immediately, but inhibits the proliferation of these cells within 72 h [27]. Thus, a lack of a simultaneous, direct cytotoxic effect on neoplastic, stromal, and especially endothelial cells in solid tumors may have contributed to the limited tumor response in our study. In fact, in this study, we could not prove that the toxin indeed binds to the tumor cells, as we only have indirect evidence. OMM cells showed positivity for immunostainings of uPA, uPAR, MMP-2, MT1-MMP, and TIMP-2. After intratumoral treatment with the anthrax toxin, necrotic areas were seen through histopathology, meaning that tumor cells were dead. In the same samples, we could see necrosis of blood vessels endothelial cells. Therefore, we argue that necrosis of OMM cells could be a direct effect of the toxin, and/or the OMM cell necrosis could result from the impairment of blood circulation due to necrosis of blood vessel walls. The dogs’ immune system may have also contributed to the results obtained in our study. The *Bacillus anthracis* toxin can stimulate production of monoclonal antibodies throughout the treatment, which may neutralize the toxin and decrease its antitumor activity. Brossier et al. (2004) [44] reported lower cytotoxicity when monoclonal antibodies were used associated with *B. anthracis* toxin in cultured macrophages and decreased lethality in mice infected with the anthrax toxin. Liu et al. (2016) [27] used immunosuppressors such as pentostatin and cyclophosphamide associated with the reengineered anthrax toxin treatment in metastatic carcinoma. The authors found that this treatment had prolonged the anti-tumor effects, which lasted 10 days after the first cycle of therapy, by blocking neutralizing antibody production. This result may explain why tumors in dogs 1 and 2 decreased until day 7 in our experiment.

## 4. Conclusions

In summary, canine OMM in our study expressed uPA, uPAr, and metalloproteinases. The re-engineered anthrax toxin created by Liu, Leppla and Bugge showed antitumor activity and no systemic effects in dogs with OMM when administered intratumorally. Dogs had partial response or stable disease, which are considered acceptable results for OMM that are very aggressive tumors in canines. Future studies should be aimed at investigating the systemic administration of anthrax toxin to treat, not only the primary tumors, but also the melanoma metastasis.

## 5. Materials and Methods

### 5.1. Animals

The study was performed in accordance with protocols approved by the Animal Ethics Committees of the Veterinary Hospital at Anhembi Morumbi University (UAM), School of Medicine of the University of São Paulo (FM-USP), and School of Veterinary Medicine and Animal Science of the University of São Paulo (FMVZ-USP), Brazil. The Animal Study Proposal Numbers and date of approval were, respectively: 00720141 Anhembi Morumbi (approved on June 27, 2014), 052/14 FM-USP (approved on July 17, 2014), 8798100314 FMVZ-USP (approved on May 14, 2014). The protocol was approved by all the Animal Ethics Committees before the the study began.

Five dogs bearing OMM were selected according to predefined criteria (Table 7). All dogs were examined at the Veterinary Hospital at Anhembi Morumbi University and had their OMM diagnosed by cytology or histopathology.

### 5.2. Reengineered Anthrax Toxin

PA, PA-U2-R200A, PA-L1-I210A, LF, and FP proteins were constructed and purified as previously described [13,19,20,28] by the Laboratory of Parasitic Diseases of the National Institute of Allergy and Infectious Diseases of the National Institutes of Health (NIH) in Bethesda, MD, USA. The modified toxin was approved by the National Sanitary Surveillance Agency (ANVISA) of the Brazilian Ministry of Health to be acquired by the Laboratory of Experimental Oncology of the Department of Pathology at the FMVZ-USP, and was kept in a freezer at −80 °C.

### 5.3. Clinical Study

Dogs received three or six intratumoral injections of 375 μg PA-U2-R200A + 375 μg PA-L1-I210A + 250 μg LF in 2 mL of PBS for one or two weeks, every other day prior to surgery. The longest and shortest tumor diameters were measured with digital calipers with dogs put under general anesthesia on days 0, 2, 4, 7, 9, 11, and 14 of study. Physical examination and blood tests (blood cell and platelet counts, packet cell volume, ALT, AST, urea, creatinine) were performed to assess the dogs’ condition during treatment with the engineered anthrax toxin. Tumor excision was performed either on day 7 or 14.

The clinical response to intratumoral treatment with the reengineered anthrax toxin was evaluated by measuring the longest tumor length and the variation of tumor volume over seven or 14 days, with day 0 the first day of toxin administration and tumor measurements taken under general anesthesia at least on days 0, 2, 4, and 7, 9, or 14 in each of the five dogs. Tumor responses were classified according to canine Response Evaluation Criteria in Solid Tumors (cRECIST) v 1.0 [29] and characterized as: Complete response (CR), disappearance of all target lesions; partial response (PR), at least 30% reduction in tumor volume; stable disease (SD), less than 30% reduction or up to 20% increase in tumor volume; and progressive disease (PD), at least a 20% increase of the initial tumor volume [29].

### 5.4. OMM Histopathology and Immunostaining

OMM samples taken from the five dogs before, and after, treatment were routinely processed for embedding in paraffin wax, and the 4 μm sections were stained with hematoxylin and eosin for diagnosis. Additional slices of the paraffin blocks were collected in salinized slides and immunostained with uPA (urokinase plasminogen activator), uPAR (uPA receptor), MMP-2 (metalloproteinase 2), MT1-MMP (membrane MMP), or TIMP-2 (metalloproteinase inhibitor) primary antibodies (Table 8). For immunohistochemistry, 4 μm slices were cut from the paraffin blocks, dewaxed in xylene, and hydrated in alcohol, followed by antigen retrieval with citrate buffer at pH 6.0 in a Dako Pascal S2800 pressure cooker (Dako Cytomation, Carpinteria, CA, USA). The slides were then washed in deionized water, the endogenous peroxidase was blocked with H_2_O_2_ solution for 30 min, followed by another washing in deionized water and phosphate buffered saline (PBS) solution. Blocking of non-specific proteins followed. The slices were then incubated with the primary antibodies (Table 8) at 4 °C for 12–18 h and washed with PBS buffer prior to the use of the polymer detection system (Histofine^®^ Simple Stain™ Max PO; Nichirei Biosciences Inc., Tokyo, Japan) and the chromogen (AEC Substrate Chromogen Ready-to-Use, Dako). The slides were counterstained with Harris hematoxylin and mounted in Permount^TM^ aqueous mounting medium (Munchen, Germany). Additional paraffin slices of the same blocks were treated with the same immunohistochemistry protocols, but without the primary antibodies, and used as negative controls.

Antibodies against Ki-67 (Table 8) were applied for immunostainings of OMM samples, collected before and after treatment with reengineered anthrax toxin, and the positive nuclei were counted in order to evaluate cell proliferation.

## Figures and Tables

**Table 1:** Canine oral mucosal melanomas (OMM) characteristics and staging in five dogs of the study.

Dog	Breed; Gender; Age; Weight (Kg)	Staging ^A^ (I-IV)	Histological Type ^B^	Initial Volume ^C^ (mm^3^)	Main Diameter (mm)	Localization
1	Yorkshire; Male; 11; 5,4	III (T2N1bM0)	Amelanotic, epithelioid	18602	50,6	Left mandible
2	Daschund; Male; 14; 7,3	III (T2N1bM0)	Melanotic, spindle	2509	36,5	Left maxilla
3	Mongrel; Female; 16; 5,7	III (T2N1bM0)	Melanotic balloon cells	8875	35,4	Right maxilla
4	Labrador; Male; 14; 33,3	III (T2N1bM0)	Melanotic, desmoplastic	7613	37,5	Pre maxilla
5	Lhasa Apso; Male; 12; 5,0	I (T1aN0M0)	Melanotic, spindle	228,3	7,7	Hard palate

^A^ Staging according to WHO; ^B^ According to Spangler & Kass, 2006 [26]; ^C^ Volume = length × width^2^ / 2.

**Table 2:** Volume reduction of OMM after reengineered anthrax toxin treatment in dogs.

Dog/Breed	Tumor Volume Day 0 (mm^3^) ^A^	Tumor Volume Day 7 or 14 (mm^3^) ^A^	% of Tumor Reduction
1 Yorkshire terrier	18602	16402 (day 14)	12%
2 Daschund	2509	1341 (day 14)	47%
3 Mongrel	8895	10754 (day 7)	20% (+)
4 Labrador retriever	7613	2847 (day 7)	63%
5 Lhasa apso	228,3	148 (day 7)	34%

^A^ Volume = length × width^2^ / 2 (Sugiura *et al.,* 1952) [28].

**Table 3:** Clinical pathology data of five dogs included in the study, before and after (**b/a)** the intratumoral inoculations of the reengineered anthrax toxin.

Dog	PCV (%) (b/a)	Leukocytes (×1000cels/μL) (b/a)	Alanine transferase (U/L) (b/a)	Alkaline Phosphatase (U/L) (b/a)	Urea (mg/dl) (b/a)	Creatinin (mg/dl) (b/a)	Platelet (×1000/mL) (b/a)	Weeks after Treatments
1	46/40	19,9/28,4	43/12	29/69	43/47	1,0/0,8	366/285	5
2	50/43	34/12,9	31/56	248/655	58/34	0,7/0,9	569/407	9
3	34/25	29,6/14,1	99/264	395/691	64/94	1,3/1,3	678/474	14
4	43/41	13,7/6,1	57/60	110/92	25/35	0,8/0,8	392/312	21
5	47/44	6,3/8,69	56/52	131/138	43/155	1,2/1,0	438/553	22

**Table 4:** Clinical response of OMM after toxin treatment in five dogs.

Dog	Breed; Gender; Age; Weight (Kg)	Histological Type ^A^	Main Diameter (mm) before Toxin Inoculation ^B^	Main Diameter (mm) after Toxin Inoculation^B^	cRECIST^B^
1	Yorkshire terrier; Male; 11; 5,4	Amelanotic, epithelioid	50,6	45	Stable disease
2	Daschund; Male; 14, 7,3	Melanotic, spindle	36,5	32,1	Stable disease
3	Mongrel; Female; 16; 5,7	Melanotic balloon cells	35,4	35,1	Stable disease
4	Labrador; Male; 14; 33,3	Melanotic, desmoplasic	37,5	32,3	Stable disease
5	Lhasa Apso; Male; 12; 5,0	Melanotic, spindle	7,7	6,7	Stable disease

^A^ According to Spangler & Kass, 2006 [26]; ^B^ Nguyen et al., 2015 [29].

**Table 5:** Presence or absence of necrosis in histopathological analysis and quantification of Ki-67 positive cells in OMM after intratumoral treatment with reengineered anthrax toxin.

Dog	Necrosis/ Endothelial Cells Necrosis after Treatment	Ki67 Positive Cells Quantification (%) before Treatment	Ki67 Positive Cells Quantification (%) after Treatment
1	Yes/no	≥19	≥19
2	Yes/no	≥19	12,2
3	Yes/yes	≥19	7
4	Yes/yes	8,2	4,6
5	Yes/yes	3,6	16

**Table 6:** Immunostainings of uPA, uPAr, MMP-2, MT1-MMP and TIMP-2 in dogs 1–5 with OMM.

Dog	uPA	uPAr	MMP-2	MT1-MMP	TIMP-2
1	+	+	-	+	+
2	+	+	+	+	+
3	+	+	+	+	+
4	+	+	+	+	+
5	+	+	+	+	+

**Table 7:** Criteria for the inclusion of dogs in the reengineered anthrax toxin clinical study.

Criteria	Ideal Condition
Histopathological or cytological diagnosis	Melanoma
Localization of the tumor	Oral cavity (mandible or maxila), and measurable with a pachimeter
Radiographic exam of the thorax	No metastasis in lungs
Ultrasound examination of abdomen	No metastasis in liver and spleen
Complete blood examination, serum biochemistry for liver and kidney function	Good general condition.

Tumor volume was calculated using the formula: volume = (length × width²)/2 [26].

**Table 8:** Antibodies used for the immunohistochemical analysis of canine OMM samples.

Antibody	Code	Mono/ Polyclonal	Mouse or Rabbit	Dilution	Subcellular Localization
uPA H140–Santa Cruz	Sc14019	Polyclonal	rabbit	1:200	Cytoplasm
uPAR–Dako	M7294	Monoclonal	mouse	1:200	Membrane
MMP-2– Abcam	Ab86607	Monoclonal	mouse	1:200	Membrane
MT1-MMP–Abcam	Ab53712	Polyclonal	rabbit	1:200	Membrane
TIMP-2–Abcam	b1828	Monoclonal	mouse	1:200	Membrane
Ki67–Dako	M7240	Monoclonal	Mouse	1:50	Nucleus
